# Changes in the faecal microbiota of horses and ponies during a two-year body weight gain programme

**DOI:** 10.1371/journal.pone.0230015

**Published:** 2020-03-19

**Authors:** Katharina Langner, Dominique Blaue, Carola Schedlbauer, Janine Starzonek, Veronique Julliand, Ingrid Vervuert

**Affiliations:** 1 Institute of Animal Nutrition, Nutrition Diseases and Dietetics, Leipzig University, Leipzig, Germany; 2 PAM UMR A 02.102, AgroSup Dijon, Université Bourgogne Franche- Comte, France; University of Maine, UNITED STATES

## Abstract

Obesity is a major health concern in many domesticated equids animals since it is related to metabolic abnormalities such as insulin dysregulation, hyperlipidaemia or laminitis. Ponies especially are known as “easy keepers” and are often affected by obesity and its related metabolic disorders. Research in the last decade indicated that the intestinal microbiota may play an important role in the development of obesity, at least in humans. Therefore, the objective of our study was to characterize changes in the faecal microbiota during a two-year weight gain programme which compared ponies and warmblood horses. For this purpose, 10 Shetland ponies and ten warmblood horses were fed a ration which provided 200% of their maintenance energy requirement over two years. Feed intake, body weight, body condition and cresty neck score were recorded weekly. At three standardized time points faecal samples were collected to characterize the faecal microbiota and its fermentation products such as short chain fatty acids and lactate. Next generation sequencing was used for the analysis of the faecal microbiota. During body weight gain the richness of the faecal microbiota decreased in ponies. Besides changes in the phylum Firmicutes in ponies that were already described in human studies, we found a decrease of the phylum Fibrobacteres in horses and an increase of the phylum Actinobacteria. We were also able to show that the phylum Fibrobacteres is more common in the microbiota of horses than in the microbiota of ponies. Therefore, the fibrolytic phylum Fibrobacteres seems to be an interesting phylum in the equine microbiota that should receive more attention in future studies.

## Introduction

Recent data from the United States and Australia indicate that 23–51% of the equine population may be overweight or obese [1–3]. This is a major concern for the horse welfare because equine obesity increases the risk for metabolic abnormalities such as insulin dysregulation (ID) and laminitis [3]. Obesity is also an important factor in the equine metabolic syndrome (EMS) that also includes other clinical signs like laminitis and ID [4].

As hindgut fermenters horses have a bacterially dominated gut microbiota, that produces short chain fatty acids and lactate through anaerobic fermentation of structural carbohydrates [5]. Its main phyla are Firmicutes and Bacteroidetes [6] but there is a high variability, influenced by factors like ration [7], age [8–10], hindgut section [11], health status [12] and individual variation [13].

Research in the last decade emphasized the role of the gut microbiota in obesity in different animal species and humans. It has been demonstrated that obese mice and humans tended to have a less diverse gut microbiota [14,15] with a relative increase of the phylum Firmicutes and a decrease of the phylum Bacteroidetes [14,16,17]. Interestingly, studies with germ free mice showed that these animals were less likely to gain weight than mice with a colonised gut [18–20]. Germ free mice whose gut was artificially colonised by a Firmicutes dominated microbiota, harvested from overweight mice, gained more weight than mice whose gut was colonised by a wild- type mouse microbiota [21].

However, in human studies where the impact of diet on the microbiota is less controlled, inconsistent results have been published [22–24].

There is an ongoing discussion about how the microbiota influences body weight (BW). A study investigating the metagenomics of a Firmicutes dominated microbiota of genetically obese mice and the Bacteroidetes dominated microbiota of their lean littermates, showed an increased capacity for energy harvest in the obese mice microbiota [21]. Enzymes that are necessary to catabolise otherwise indigestible polysaccharides were elevated in the obese mice microbiota. In the caecum of obese mice the main fermentation products acetate and butyrate, which contribute to the host’s energy supply, were also enriched [21]. But other authors working with a different mouse strain showed lower caecal concentrations of SCFA in obese mice [25]. In horses no differences in the faecal SCFA concentrations of different weight groups have been demonstrated [9,26].

There has been an increasing interest in the equine microbiota and its link to obesity. However, to the author’s knowledge there are only three studies that compared the equine microbiota of different weight groups. In contrast to studies in mice [14] and humans [15,27] the studies in horses showed a higher diversity in the faecal microbiota of obese individuals [9,28]. These results were also contrary to findings from Elzinga et al. [29] in obese EMS horses, that showed a less diverse faecal microbiota than a control group.

Regarding the distribution of the faecal microbiota phyla, differing results were found in studies conducted by Morrison et al. (2018) [9] and Biddle et al. (2018) [28] for the phylum Bacteroidetes. Biddle et al. (2018) who studied mainly horses fed on different amounts of hay, grass and concentrate showed a decrease of Bacteroidetes in the faecal microbiota of obese horses. In contrast, Morrison et al. (2018) [9] fed a standardised hay diet and these authors found a higher abundance of Bacteroidetes in the faecal microbiota of obese Welsh ponies. On the other hand, both authors showed that the phylum Firmicutes increased in the microbiota of obese horses. The increase in the phylum Firmicutes in the microbiota was also described in obese humans [30,31]. Furthermore, a higher abundance of the phylum Actinobacteria and a lower abundance of the phylum Fibrobacteres was detected in obese horses by Morrison et al. (2018). In contrast, Shepard et al. (2014) [26] did not find any significant changes in the microbiota phyla of non-obese and obese horses fed a hay diet.

Ponies are known as “easy keepers” that have a lower energy requirement per unit of metabolic body weight than horses [32]. Therefore ponies are more prone to obesity [3] and related disorders such as laminitis [33–35]. A recent study also demonstrated that the metabolic function of the liver is more affected by body weight gain in ponies than in horses [36].

The aim of our study was to investigate changes in the faecal bacteria and the fermentation products between horses and ponies during a two- year BW gain programme caused by excessive energy intake. We hypothesized that the bacteria of both horses and ponies would become less diverse with an increase of the phylum Firmicutes and a decrease in the phylum Bacteroidetes during the body weight gain period. Due to shifts in the bacterial phyla we also expected changes in the SCFA and Lactate pattern.

## Material and methods

### Animals and management

As a part of a larger project about the metabolic differences between horses and ponies during a two-year BW gain programme [37], ten Shetland pony and ten Warmblood horse geldings owned by the Institute of Animal Nutrition, Nutrition Diseases and Dietetics of the University of Leipzig were housed and fed according to a standardized protocol. The mean (± SD) age was 6 (± 3) years for the Shetland ponies and 10 (± 3) years for the horses. Before the onset of the study, insulin sensitivity was evaluated by a combined glucose and insulin test according to Eiler et al [38] and Pituitary Pars Intermedia Dysfunction (PPID) was excluded via determination of ACTH after 8 hours of fasting [39]. An experienced clinician confirmed the absence of clinical or radiological signs of previous or acute laminitis in all animals. The animals were bedded on straw in individual box stalls and were turned out onto a dry lot for approximately 5 hours a day. The project was approved by the Ethics Committee for Animal Right Protection of the Leipzig District Government (No. TVV 32/15) in accordance with German legislation for animal rights and welfare.

The experimental study was conducted over two years. During an adaption period, ponies and horses received meadow grass hay and a commercial mineral supplement to meet or exceed energy and nutrient requirements during maintenance according to the Society of Nutrition Physiology (GfE 2014) [40]. The basal health status of the animals was assessed at the beginning of the trial (t0). Following this first data collection animals underwent a feeding period receiving 180% of their individual maintenance metabolizable energy requirements according to GfE (2014) [40]. Seventy percent of the energy intake was supplied by hay and 30% was provided by a concentrate (nutrient composition based on dry matter (DM): crude protein: 11.7%, crude fat: 12.8%, crude fibre: 9.9%, crude ash: 7.1%, metabolizable energy: 11.75 MJ/kg DM).

Faecal samples were taken after a five-month adaption period to the experimental diet (t1). In addition, faecal samples were collected twelve (t2) and twenty-four (t3) months after the start of the trial (t0). Between the first (t1) and the second (t2) sampling point the energy intake was increased to 200% supplied by a ration consisting out of 60% hay and 40% concentrate. There was a change in concentrate between the first and second sampling point due to production problems by the manufacturer (nutrient composition based on DM of the new concentrate: crude protein: 13.4%, crude fat: 14.4%, crude fibre: 9.78%, nitrogen-free extract: 54.3%, metabolizable energy: 14.09 MJ/kg DM). The nutrition composition was analysed monthly for the hay and yearly for the concentrate. Crude nutrients were analysed by Weende analysis. Starch content was determined polarimetric and sugar content with the Luff- Schoorl method. The analysis of feedstuff was performed according to the official collection of methods from the European Union [41]. Neutral detergent fibre was determined based on amylase treatment and incineration (aNDFom). The analysed values were used for the calculation of the energy content. Energy intake was adapted monthly to the current BW. Feed refusals were recorded on a weekly basis.

### Scaling, Body Condition Score (BCS) and Cresty Neck Score (CNS)

Body weight, BCS and CNS were measured weekly. An electronic large animal scale (scale system Iconix FX 1, Texas Trading, scale precision: 0.5 kg) was used to determine BW. BCS was assessed on a scale from 0 to 5 as described by Carrol and Huntington (1988) [42]. The CNS was evaluated on a scale from 0 to 5 according to Carter et al (2009) [43]. Two trained evaluators performed both scores and the mean of their evaluation was calculated for data analyses.

### Faecal sampling

Faecal samples were taken using a collection bag that was attached around the anus with tape three hours after the morning meal. Directly after the collection three Eppendorf tubes (Eppendorf, Hamburg, Germany) were filled with 1 g of faeces and gradually frozen from -20°C to -80°C pending DNA extraction. In addition, 10 g of faeces were mixed with 10 ml of distilled water and centrifuged for 10 minutes at 6000 rpm. From the supernatant 1ml was transferred in Eppendorf tubes for lactate analysis. For the SCFA analysis 1 mL of the supernatant was transferred in tubes containing 0.1 ml of a buffer composed of 4.25% H_3_PO_4_ and 1% HgCl_2_. Freezing protocol was the same as described above.

### SCFA analysis

For the SCFA analysis the samples were mixed with 100 μl of ethanol and centrifuged for 10 minutes at 6000rpm (5417c, Eppendorf, Hamburg, Germany). In the filtrated supernatant the concentration of the short chain fatty acids (SCFA) acetate (C2), propionate (C3), isobutyrate (IC4), butyrate (C4), isovalerate (IC5) and valerate (C5) were measured by gas liquid chromatography (Clarus 500, Perkin Elmer, Waltham, US) [44]. All samples were measured in duplicate and the mean coefficient of variation was 4.8% for C2, 3.07% for C3, 3.15% for IC4, 2.84% for C4, 1.77% for IC5 and 8.87% for C5.

### Lactate analysis

For lactate analysis the samples were deproteinized in 100 μl perchloric acid. Subsequently the enzymatic colorimetric lactate analysis was performed using a D- and L- Lactic Acid Kit (Megazyme International, Ireland) according to the manufacturer’s instructions. The quantity of the lactate was measured at a wavelength of 340 nm in a 96 well plate with a MRX^e^ tc microplate reader (Dynex, Lincoln, United Kingdom). All samples were measured in duplicate and the mean coefficient of variation was 1.57%.

### DNA extraction and sequencing

The total DNA was extracted from 0.25g of faeces using the bead- beating method described by Yu and Morrison (2004) [45]. The quantity of the extracted DNA was measured using a Biophotometer 6131 (Eppendorf, Hamburg, Germany) and the purity was assessed by the calculation of the A260/280 ratio to screen the samples for protein contamination [46]. A260/280 ratios between 1.5 and 2.0 were accepted for the DNA. Afterwards a PCR was performed to amplify the V3-V4 region of the 16S rRNA. The PCR Mix consisted of 0.4 μl of dNTP 25mM, 1.25 μl of every primer (Table 1), 0.5 μl of Taq Polymerase (D 7442, Sigma-Aldrich, Saint Louis, USA), 5 μl of the corresponding Taq buffer, 39.1 μl of water and 10 ng of DNA for each sample. The samples were diluted with water 1 to 4 or 1 to 64, dependent on the biophotometer results. Then 5 μl of each diluted sample were added to the PCR Mix. The amplification was performed with a C 100 touch thermocycler (BioRad, Hercules, USA) using the following programme: 94°C for 60 seconds for the initial denaturation, followed by 30 cycles of 94°C for 60 s (denaturation), 65° C for 60 s (annealing) and 72°C for 60 s (elongation) and a final elongation at 72°C for 10 minutes. For visualization 4 μL of each PCR product were pipetted into a well of a 2% agarose gel. Electrophoresis of the gel was performed for 1h15min at 45°C and 70 V on a Mupid One system (ADVANCE co. Ldt., Tokyo, Japan). Afterwards the gel was stained with gel red (Biotium, Fremont, US) and the quality of the amplicons was checked using ultraviolet light. After purification of the first PCR with PCR clean magnetic beads a second PCR was performed to ligate Illumina adapters and an index that allowed the identification of the samples. The PCR mix was similar to the first PCR with only the primers changed (Table 1). The PCR conditions were identical to the previous PCR, but 12 cycles were performed instead of 30. Afterwards PCR products were purified and sequenced with an Illumina MiSeq run of 250 base paired-ends following the manufacturer’s instructions (Illumina Inc., San Diego, CA). The quality of the run was assessed with control libraries generated with the PhiX virus (Illumina PhiX control; Illumina Inc., San Diego, CA).

**Table 1:** Sequences of the primers used for the DNA amplification.

Forward Primer 1 PCR1F343 [47]	5´ CTTTCCCTACACGACGCTCTTCCGATCTACGGRAGGCAGCAG
Reverse Primer 1 PCR1R784 [47]	5´ GGAGTTCAGACGTGTGCTCTTCCGATCTTACCAGGGTATCTAATCCT
Forward Primer 2 [47]	5´ AATGATACGGCGACCACCGAGATCTACACTCTTTCCCTACACGAC
Reverse Primer 2 [47]	5´ CAAGCAGAAGACGGCATACGAGAT-Index-GTGACTGGAGTTCAGACGTGT

### Data analysis

The sequencing data was processed on the Galaxy signenae workbench provided by INRA Toulouse using the Find Rapidly OTUs with Galaxy Solutions (FROGS) pipeline. First the sequences were cleaned through elimination of sequences shorter than 380 base pairs or longer than 490 base pairs, and those containing an unidentified base or not containing one of the primer sequences. Chimeras were identified using VSEARCH [48] and removed from further analysis. The remaining sequences were aligned in clusters with SWARM [49]. Clusters that contained only one sequence were excluded from further analysis and the remaining clusters were grouped in operation taxonomic units (OTUs). The taxonomic assignment of the OTUs was performed with the Basic Local Alignment Search Tool (BLAST) provided by the National Centre for Biotechnology Information database. The Shannon index and observed richness were calculated on the Galaxy signenae workbench. The further grouping of the data and the calculation of the relative abundance of OTUs were conducted with Excel (Office 365, Microsoft, Redmond, US).

The Excel software programme was also used for the calculation of the coefficient of variation (CV) for crude protein (CP), crude lipid (CL), crude fibre (CF), neutral detergent fibre (aNDFom), sugar and starch intake to determine variations in nutrition intake. The CV was also calculated for the DM intake of hay during the study period. For the analysis of the CV the data were grouped in months for horses and for ponies. The CV was calculated by the mean nutrition intake for the last two months prior to sampling for horses and ponies at each sampling point and between the different sampling points.

The statistical analyses were performed using the commercial software package STATISTICA (Version 12, StatSoft GmbH, Hamburg, Germany). Data were analysed for normal distribution by using the Shapiro-Wilks test. BCS, CNS, SCFA, lactate and microbial community were analysed using non-parametric tests. Friedman´s ANOVA and Wilcoxon signed rank test with Bonferroni correction was performed factoring the effects of time. For breed related differences Mann-Whitney-U test was used. Data are presented as medians and 25 and 75 percentiles. Statistical significance was accepted at P < 0.05.

## Results

One pony and one horse developed an episode of laminitis during the second year of BW gain. Additionally, one pony developed hyperlipemia (serum triglycerides: 14.4 mmol/L) at the end of the second year of excessive energy intake. Faecal samples were collected from these individuals only after clinical improvement. Between the second and the third sample collection one of the horses had to be euthanized due to a severe episode of colic. Therefore only 59 faecal samples (30 ponies, 29 horses) were collected at three different time points (t1, t2 and t3).

CV of CP, CL, CF, aNDFom, sugar and starch intake showed a variation between 4% and 13% between the two breeds prior to each sampling point (S1 Table). The variation between t1, t2 and t3 prior to sampling ranged from 10% to 22%. The highest variation between sampling points was recorded for CL between the two months prior to t1 and t2 where the concentrate was changed due to production problems by the manufacturer.

### BW, BCS and CNS

Between t1 and t2 a significant increase in BW was recorded for ponies (p = 0.005) and horses (0.005. Between t2 and t3 only a numeric increase in BW was seen in both breeds (Table 2). CNS and BCS rose significantly between all three time points for horses and ponies to a median of 3.5 for CNS and 4.8 for BCS.

**Table 2:** BW, BCS and CNS for horses and ponies. Data are presented as medians and 25/75 percentiles in brackets.

Variable	Breed	t1	t2	t3
BW (kg)	Ponies	110^a^^#^ (103/119)	134^b^^#^ (126/146)	139^b^^#^ (132/158)
	Horses	612^a^* (578/650)	689^b^* (554/730)	696^b^* (678/732)
BCS (0–5)	Ponies	4^a^ (3.9/4.0)	4.6^b^ (4.4/4.7)	4.8^c^ (4.7/5.2)
	Horses	4^a^ (3.9/4.0)	4.6^b^ (4.5/4.6)	4.8^c^ (4.7/4.9)
CNS (0–5)	Ponies	2^a^ (1.8/2.3)	2.8^b^ (2.5/3.0)	3.5^c^ (3.3/4.0)
	Horses	2^a^ (2.0/2.3)	2.9^b^ (2.8/3.0)	3.5^c^ (3.5/4.0)

a, b, c medians with different superscript letters differ significantly within a row (p < 0.05)

*, # medians with different superscript symbols differ significantly within a column (p < 0.05)

### Sequencing metrics

From the 59 sequenced faecal samples 1184086 (mean 20069.2 /sample) raw sequences were obtained. After quality filtering during the bioinformatic processing 635820 (mean 10776.6 /sample) sequences remained. These sequences were assigned to 2219 OTUs that were grouped into 11 phyla, 19 classes, 29 orders and 62 families, 145 genera and 36 species.

### Bacterial community composition

**Fig 1:**
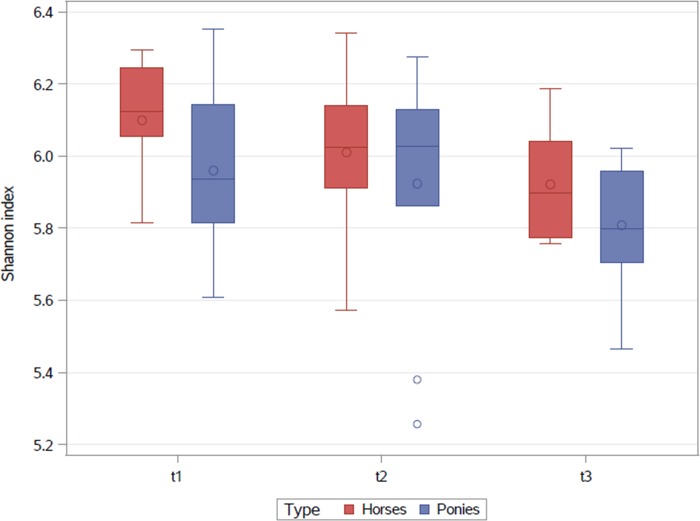
Shannon index at the three sampling points (t1-t3) for horses and ponies. The interquartile range is indicated by the box. The dot inside the box indicates the mean and the line the median. The whiskers represent the lower and upper extremes. Outliers are indicated as single dots.

**Fig 2:**
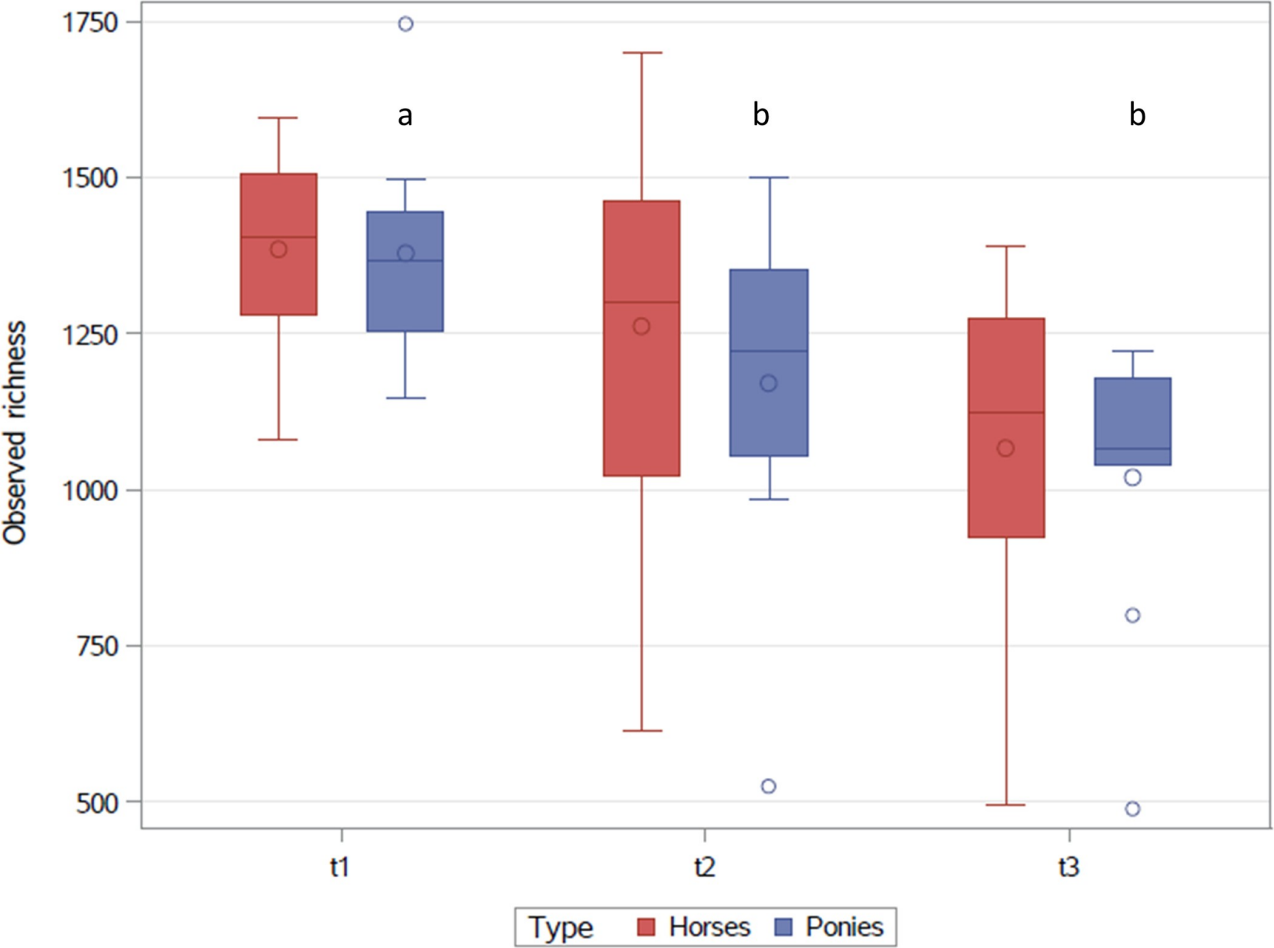
Observed richness at the three sampling points (t1-t3) for horses and ponies. The interquartile range is indicated by the box. The dot inside the box indicates the mean and the line the median. The whiskers represent the lower and upper extremes. Outliers are indicated as single dots.

The alpha diversity, described by the Shannon index showed no significant differences between the three timepoints or breeds (Fig 1). During the BW gaining programme a significant decrease in richness was recorded for the ponies between t1 and t2 (p = 0.025) and t1 and t3 (p = 0.005), but no significant changes were seen for horses (Fig 2)

From the 11 phyla, 19 classes, 29 orders, 62 families, 145 genera and 36 species identified, only 6 phyla, 7 classes, 7 orders and15 families had a median relative abundance over 0.4% of the faecal microbiota. 24.7% of the genera and 97.2% of the species are unknown. 1.1% of the genera and 1% of the species could not be identified because the pipeline found various affiliations. Due to the high rate of unidentified sequences the genera and species were excluded from the further analysis to avoid bias. The most abundant phyla were Firmicutes (58.2%) followed by Bacteroidetes (36.5%), Spirochetes (2.46%), Fibrobacteres (1.18%), Proteobacteria (0.69%) and Actinobacteria (0.63%). At t3 one sample taken from a horse showed a very high abundance (22.76%) of the phylum Actinobacteria. In general, the phylum Actinobacteria ranged between 0.12% and 2.58%. Therefore, we decided to exclude this sample from further analysis, as it was probably contaminated.

### Faecal microbiota of horses and ponies

The phylum Fibrobacteres was more abundant in the faecal microbiota of horses than in the faecal microbiota of ponies at timepoint t3 (p = 0.026) (Table 3 and S2 Table). The significant higher relative abundance for horses at t3 was also seen for the corresponding class, order and family (S3–S5 Tables). For the phylum Proteobacteria a significant higher relative abundance was found at t2 in the faeces of ponies than in the faeces of horses (p = 0.01).

**Table 3:** Phyla, classes, orders and families in faeces of horses and ponies with a median relative abundance over 0.4% with significant changes between the three sampling points or between breeds. Data are presented as medians and 25/ 75 percentiles in brackets.

Phylum	Breed	t1	t2	t3
Firmicutes	Horses	55.7 (52.3/59.8)	57.2 (55.5/62.2)	58.5 (55.5/62.9)
	Ponies	54.0^ab^ (51.9/62.9)	58.5^a^ (53.2/63.8)	62.8^b^ (57.7/65.7)
Fibrobacteres	Horses	1.90^a^ (1.05/2.45)	0.98^b^ (0.85/1.29)	1.11^b^^#^ (0.59/1.84)
	Ponies	0.78 (0.51/2.31)	0.79 (0.65/1.70)	0.38* (0.09/0.88)
Proteobacteria	Horses	0.34 (0.31/0.69)	0.61^#^ (0.52/0.92)	0.52 (0.43/0.99)
	Ponies	0.60^a^ (0.37/0.85)	1.14^b^* (0.81/1.45)	0.43^a^ (0.27/0.70)
Actinobacteria	Horses	0.37^a^ (0.30/0.49)	0.34^ab^ (0.30/0.43)	0.88^b^ (0.54/1.31)
	Ponies	0.33^a^ (0.31/0.49)	0.39^a^ (0.33/0.59)	0.77^b^ (0.67/1.25)
**Classes**				
Fibrobacteria	Horses	1.90^a^ (1.06/2.45)	0.98^b^ (0.85/1.29)	1.11^b^^#^ (0.59/1.84)
	Ponies	0.78 (0.51/2.31)	0.80 (0.65/1.70)	0.38* (0.09/0.88)
Bacilli	Horses	0.39^a^ (0.23/0.66)	0.42^a^ (0.27/0.61)	1.21^b^ (0.81/2.37)
Coriobacteriia	Horses	0.37^a^ (0.29/0.49)	0.34^ab^ (0.30/0.43)	0.80^b^ (0.52/1.29)
	Ponies	0.33^a^ (0.30/0.49)	0.39^a^ (0.33/0.59)	0.77^b^ (0.67/1.25)
Negativicutes	Horses	0.31 (0.12/0.38)	0.57 (0.41/0.70)	0.49 (0.39/0.57)
	Ponies	0.32^a^ (0.27/0.64)	0.81^b^ (0.41/1.24)	0.39^a^ (0.23/0.84)
**Orders**				
Fibrobacterales	Horses	1.90 (1.06/2.45)	0.98 (0.85/1.29)	1.11^#^ (0.59/1.84)
	Ponies	0.78^a^ (0.51/2.31)	0.80^b^ (0.65/1.70)	0.38^a^* (0.09/0.88)
Coriobacteriales	Horses	0.37^a^ (0.30/0.49)	0.34^ab^ (0.29/0.43)	0.80^b^ (0.52/1.29)
	Ponies	0.33^a^ (0.30/0.49)	0.39^a^ (0.33/0.60)	0.77^b^ (0.67/1.25)
Selenomonadales	Horses	0.31 (0.12/0.38)	0.57 (0.41/0.70)	0.49 (0.39/0.57)
	Ponies	0.32^a^ (0.27/0.64)	0.81^b^ (0.41/1.24)	0.39^a^ (0.23/0.84)
**Families**				
Ruminococcaceae	Horses	25.0^a^ (22.5/29.1)	24.2^a^ (22.7/31.9)	21.4^b^^#^ (19.0/22.6)
	Ponies	24.5 (23.9/32.2)	27.2 (24.1/33.9)	25.2* (20.9/26.1)
Lachnospiraceae	Horses	23.9^a^ (20.5/25.6)	22.9^ab^ (20.9/26.4)	31.3^b^ (25.9/32.5)
	Ponies	22.7 (20.7/23.3)	22.7 (20.8/25.0)	26.8 (24.0/28.6)
Rikenellaceae	Horses	7.15 (5.33/7.65)	5.63 (4.19/6.39)	4.80^#^ (4.18/6.17)
	Ponies	7.40^ab^ (4.51/8.17)	5.58^a^ (4.77/5.93)	6.80^b^* (5.56/9.50)
F082	Horses	2.66^a^^#^ (1.81/2.84)	4.46^a^ (2.13/9.44)	1.50^b^^#^ (1.10/1.88)
	Ponies	6.34* (4.09/9.94)	5.67 (3.29/7.00)	5.16* (2.65/5.74)
Bacteroidales UCG-001	Horses	1.14 (0.94/1.84)	0.90 (0.62/1.39)	0.60^#^ (0.52/0.93)
	Ponies	1.22 (1.02/1.64)	0.90 (0.75/1.13)	1.21* (1.03/142)
Fibrobacteraceae	Horses	1.90^a^ (1.06/2.45)	0.98^b^ (0.85/1.29)	1.11^b^^#^ (0.59/1.84)
	Ponies	0.78 (0.51/2.31)	0.80 (0.65/1.70)	0.38* (0.09/0.88)
Muribaculaceae	Horses	0.75 (0.61/1.17)	1.55 (1.10/1.74)	1.24 (0.84/2.81)
	Ponies	0.93^a^ (0.47/1.93)	1.62^b^ (1.13/2.64)	1.14^a^ (0.72/1.57)
Bacteroidales RF16 group	Horses	0.81^a^ (0.59/1.00)	0.45^ab^ (0.28/0.63)	0.29^b^ (0.13/0.50)
	Ponies	0.62^a^ (0.36/0.89)	0.40^ab^ (0.35/0.49)	0.17^b^ (0.14/0.28)
Veillonellaceae	Horses	0.31 (0.12/0.38)	0.57 (0.41/0.70)	0.49 (0.39/0.57)
	Ponies	0.32^a^ (0.27/0.64)	0.81^b^ (0.41/1.24)	0.39^a^ (0.23/0.84)

a, b medians with different superscript letters differ significantly within a row (p < 0.05)

*, # medians with different superscript symbols differ significantly within a column (p < 0.05)

At the family level Ruminococcaceae (p = 0.033), Rikenellaceae (p = 0.026), F082 (p = 0.002) and Bacteroidales UCG-001 (p = 0.041) had higher concentrations in the faeces of ponies than in the faeces of horses at t3 (Table 3 and S5 Table). For the family F082 the higher relative abundance in the pony faeces was also seen at timepoint t1 (p = 0.003).

### Changes in the faecal microbiota during the two-year weight gain programme

The phylum Firmicutes increased significantly between t2 and t3 in the faecal microbiota of ponies (p = 0.047) while no changes were seen for the horses. While the phylum Fibrobacteres decreased between t1 and t2 in the faecal microbiota of horses (p = 0.028), the phylum Proteobacteria in the faecal microbiota of ponies showed a significant increase between t1 and t2 (p = 0.005) followed by a significant decrease between t2 and t3. Actinobacteria increased between t2 and t3 in ponies (p = 0.017) and between t1 and t3 in horses (p = 0.02). More information about significant changes in classes, orders and families during the two-year weight gaining programme are presented in the supporting information (S2, S3, S4 and S5 Tables).

### Faecal SCFA and lactate

The most abundant SCFA in the faeces wereC2 followed by C3, C4, IC5, IC4 and C5. Higher concentrations in the faeces of horses than in the faeces of ponies were found for total SCFA at t3, C2 at t2, C3 at t3, IC5 at t3 and IC4 at t1 and t2 (Table 4). Between the different timepoints no significant changes were determined in the faecal concentration of SCFA in ponies and horses. At t2 the total lactate concentration was higher in the faeces of ponies than in the faeces of horses (Table 4). During the feeding programme a significant increase in lactate was analysed for horses and ponies between t1 and t2 followed by a significant decrease between t2 and t3 in horse faeces.

**Table 4:** Total SCFA, C2, C3, IC4, IC5 and lactate concentrations (mmol/L) in the faeces of horses and ponies. Data are presented as medians and 25/ 75 percentiles in brackets.

Variable	Breed	t1	t2	t3
**Total SCFA (mmol/L)**	Ponies	19.1 (16.2/23.9)	15.9 (13.3/19.3)	16.3^#^ (11.9/21.4)
	Horses	20.7 (20.4/29.6)	18.7 (17.1/24.2)	24.9* (17.2/26.9)
**C2 (mmol/L)**	Ponies	12.4 (11.7/16.3)	10.5^#^ (8.9/11.8)	10.9 (8.1/14.6)
	Horses	17.5 (14.0/19.9)	12.6* (11.9/16.2)	16.5 (11.6/18.3)
**C3 (mmol/L)**	Ponies	4.29 (3.11/5.27)	3.78 (2.57/4.39)	3.8^#^ (2.57/4.46)
	Horses	4.76 (4.32/6.89)	4.12 (3.78/5.41)	5.68* (4.60/6.35)
**C4 (mmol/L)**	Ponies	1.19 (1.08/1.36)	1.08 (0.91/1.25)	0.94 (0.68/1.53)
	Horses	1.25 (1.08/1.65)	1.25 (0.97/1.36)	1.53 (1.02/1.71)
**IC4 (mmol/L)**	Ponies	0.28^#^ (0.23/0.34)	0.23^#^ (0.23/0.23)	0.26 (0.23/0.34)
	Horses	0.43* (0.34/0.51)	0.37* (0.34/0.46)	0.34 (0.28/0.46)
**C5 (mmol/L)**	Ponies	0.20 (0.20/0.20)	0.17 (0.10/0.20)	0.17 (0.10/0.29)
	Horses	0.20 (0.15/0.29)	0.20 (0.20/0.25)	0.20 (0.15/0.20)
**IC5 (mmol/L)**	Ponies	0.39 (0.34/0.49)	0.29^#^ (0.20/0.29)	0.29 (0.20/0.54)
	Horses	0.54 (0.40/0.67)	0.44* (0.39/0.54)	0.39 (0.39/0.49)
**Lactate (mmol/L)**	Ponies	1.06^a^ (0.81/1.19)	0.71^b^^#^ (0.56/0.81)	0.67^b^ (0.64/0.87)
	Horses	0.92^a^ (0.70/1.14)	0.50^b^* (0.43/0.64)	0.77^a^ (0.69/0.91)

a, b medians with different superscript letters differ significantly within a row (p < 0.05)

*, # medians with different superscript symbols differ significantly within a column (p < 0.05)

## Discussion

Due to the fact that ponies are more prone to obesity [2] and related diseases like laminitis [34] we investigated differences in the microbiota of horses and ponies during a two year period of excessive energy intake. To the authors knowledge this is the first paper analysing the distinctions in the faecal microbiota of horses and ponies. At present only data of the human gut microbiota revealed differences between ethnic groups [50,51]. However, unlike our animal experiment the studied human populations consumed different diets and stayed in different environments. Therefore, the impact of genetic, dietetic and environmental influences on the gut microbiota still remains open [50,51].

To the authors knowledge, our study is the first one investigating changes in the equine microbiota during a period of long-term body weight gain. This study design leads to the strong advantage that each individual was used as its own control. Other studies investigating differences of the microbiota between lean and obese horses always compared groups of different individuals [9,26,28]. It has been shown that even similar housing and feeding conditions for a six week period were not able to eliminate individual microbiota differences [13]. The large individual differences restrict the interpretation of results obtained from different individuals. The present experimental design included a two-year controlled feeding period to exclude any influences on the microbiota from different feeding regimes or management conditions. Other studies that examined the equine microbiota at different body conditions either had no standardized feeding protocol [28] or established a controlled feeding scheme for only one month or two weeks prior to faecal sampling [9,26].

During our biannual study the animals consumed 180–200% of their maintenance energy requirement according to the Society of Nutrition Physiology (GEH 2014) [40]. The major weight gain took place between t1 and t2 while only a non-significant increase in BW was recorded between t2 and t3 despite the same hypercaloric intake. In contrast CNS and BCS increased significantly in the second year of the feeding period. One explanation might be related to a loss in muscle mass due to a low physical activity during periods of turnout and increased body fat mass. However, the reasons for these findings remain open as muscle metabolism was not evaluated in this study. The lack in knowledge about muscle metabolism in the state of increasing obesity should be addressed in future studies.

During the BW gain programme faecal richness decreased in ponies. These results are in contrast to findings of Biddle et al. (2018) who found a higher richness in the faecal microbiota of obese horses. However, no changes in the faecal diversity indices were found during weight gain unlike previous data reported in humans [15] and horses [9,28]. This discrepancy might be due to the fact that most authors studied groups of different individuals and various diets in humans [15] and horses [28]. Only Morrison et al. (2018) [9] established a controlled feeding scheme one month prior to faecal sampling where the horses received a hay based ration.

In agreement with results obtained in the faecal microbiota of obese humans [30], BW gain in ponies was correlated with a significant increase in the phylum Firmicutes. Similar results have been obtained by Biddle et al. (2018) in the faecal microbiota of equines consuming varying types of forages and concentrates and by Morrison et al. (2018) who studied Welsh ponies fed a standardised hay diet. In contrast to our results, Morrison et al. (2018) and Biddle et al. (2018) found also significant changes in the phylum Bacteroidetes. In accordance with our results, Shepherd et al. (2014) did not detect any changes in the phylum Bacteroidetes in hay fed horses with various body condition scores. But unlike to our study, Shepherd et al. (2014) detected no significant changes in the phylum Firmicutes.

We also demonstrated that BW gain was associated with an increase of the phylum Actinobacteria in both breeds and a decrease of Fibrobacteres in the horse´s microbiota. These findings are in accordance with results obtained by Morrison et al. (2018) [9]. Between t1 and t2 the phylum Proteobacteria rose significantly in ponies and showed a significant drop between t2 and t3 while it remained unchanged in horses. From studies investigating the influence of a high fat ration (8.3% fat) on the faecal microbiota of horses, it is known that a high fat ration may lead to an increase of the phylum Proteobacteria [8]. Since we recorded a 22.2% increase in crude lipid intake between t1 and t2 a dietary influence on the phylum Proteobacteria cannot be excluded. From our data the reasons for the changes in the phylum Proteobacteria in ponies but not in horses are not fully understood. Since the small intestine is the main site of fat absorption [32], and the fat intake through the ration was moderate (4.4–4.7% of dry matter intake), significant effects on the hindgut microbiota are probably of minor importance. But we cannot exclude a lower precaecal fat absorption capacity in ponies than in horses.

It has been reported that a high fat intake of 14.8% dry matter reduced the digestibility of fibre probably through a reduction of the cellulytic activity in the hindgut microbiota [52] whereas a low fat intake below 10% of dry matter did not influence fibre digestibility in horses [53]. In consequence, it seems unlikely that our feeding regime with a fat intake of only 4.4–4.7% dry matter influenced fibre digestibility in the large intestine but nevertheless we cannot fully exclude an influence of the moderate changes in fat intake on the equine microbiota.

Regarding the microbial fermentation products, we could not detect any significant changes in the faecal SCFA concentrations in both breeds during weight gain. These results are in accordance with findings obtained by Shepherd et al. (2014) [26] and Morrison et al. (2018) [9] who did not find any significant differences in the faecal concentrations of SCFA in obese and lean horses.

One major difference between the microbiota of horses and ponies was a higher relative abundance of the phylum Fibrobacteres in horses at t3 that could be traced over all taxonomic categories down to the family Fibrobacteraceae. In general, a higher abundance of fibrolytic family is related to a high intake of fibre (e.g. crude fibre or neutral detergent fibre (aNDFom)), but this issue could be excluded in our study as the coefficient of variation in crude fibre and NDF intake was ≤ 10% at all sampling points between horses and ponies. In horses, the increase in the family Fibrobacteraceae may result in a better fibre fermentation, as Fibrobacteraceae is one of the fibrolytic families in the equine hindgut which are necessary for the degradation of plant cell walls [54]. These findings of a higher fibrolytic capacity in horses are in accordance with a higher digestibility of organic matter in Standardbred horses compared to Icelandic ponies fed an early cut haylage [55]. Another significant difference between horses and ponies was the higher expressions of the phylum Proteobacteria in the pony microbiota at t2. These findings are in accordance with Steelman et al. (2012)[56] who found a higher abundance of the order Burkholderiales belonging to the phylum Proteobacteria in one pony compared to a population of horses under different housing and feeding conditions [56].

In our study horses had higher concentrations of total SCFA at t3, C2 at t2, C3 at t3, IC5 at t3 and IC4 at t1 and t2 in the faeces than ponies. These results are in accordance with Jensen et al. (2010) [57] who showed that Danish warmbloods have higher faecal C3 and IC4 concentrations than Icelandic ponies when both groups received a diet consisting of sugar beet pulp, oats and haylage.

A limiting factor of our study is related to the identification of the genera. Only 1.8% of the analysed species and 74.2% of the genera were identified. Therefore, we can only draw limited functional conclusions from the generated sequencing data since the higher taxonomic categories often combine different functional groups. This is a main limitation in sequencing studies in the equine microbiota [12,58].

The analysis of faecal samples rather than samples obtained from the different sections of the gastrointestinal tract is a limitation of our study. Due to feasibility and animal welfare guidelines in most equine studies faeces have been used as a tool to describe the gut microbiota [9,13,26,28]. A correlation between dietary changes in the caecal, colonal and faecal microbiota was demonstrated by Grimm et al. (2017) [59]. But nevertheless the faecal microbiota represents at least the hindgut distal to the pelvic flexure[60].

Another limitation of our study is the fact that we only analysed faecal samples at three timepoints during the two-year study period. However, we conducted a standardised management and feeding protocol over two years. Furthermore, horses and ponies were used as their own control. Other authors already described a high stability in the faecal microbiota of individuals under identical management and feeding conditions for at least three months [13].

Reasons for equivocal results in studies comparing the microbiota of lean and obese horses might be related to differences in body weight classification and diet related differences. Similar to our study, the faecal microbiota of different individuals was compared. Biddle et al. (2018) [28] compared three different weight groups of horses while Morrison et al. (2018) [9] and Shepard et al. (2014) [26] compared an obese group of horses with a nonobese group of animals. In contrast to these study protocols, each individual horse and pony was used at its own control in our study.

In conclusion BW gain seemed to change the composition of the equine microbiota in similar ways as already described. Comparing horses and ponies we found a higher abundance of the phylum Fibrobacteria at t3 and a lower abundance of Proteobacteria at t2 in the faecal microbiota of horses. We also found higher concentrations of different SCFA in the faeces of horses at these timepoints. The changes in the fermentation profile may have functional consequences. In order to gain more knowledge about the functional consequences of the changes in the microbiota during weight gain further research has to be conducted.

## Supporting information

S1 Table Coefficient of variation for the nutrient intake in horses and ponies two months prior to sampling point t1, t2, t3 and in between the three sampling points (CP: crude protein, CL: crude lipid, CF: crude fibre, aNDFom: neutral detergent fibre). Data shown as %.
(DOCX)

S2 Table Relative abundance of Phyla in the faeces of horses and ponies with an overall median relative abundance over 0.4% at the three sampling points presented as medians and 25/ 75 percentiles in brackets. (DOCX)

S3 Table Relative abundance of Classes in the faeces of horses and ponies with an overall median relative abundance over 0.4% at the three sampling points presented as median and 25/ 75 percentiles in brackets. (DOCX)

S4 Table Relative abundance of Orders in the faeces of horses and ponies with an overall median relative abundance over 0.4% at the three sampling points presented as median and 25/ 75 percentiles in brackets. (DOCX)

S5 Table Relative abundance of Families in the faeces of horses and ponies with an overall median relative abundance over 0.4% at the three sampling points presented as median and 25/ 75 percentiles in brackets. (DOCX)

S1 Data (XLSX)

S2 Data (XLSX)

S3 Data (XLSX)

S4 Data (XLSX)

S5 Data (ODS)

S6 Data (XLSX)

S7 Data (XLSX)

S8 Data (ODS)

S9 Data (XLSX)

## References

[1] Thatcher CD, Pleasant RS, Geor RJ, Elvinger F. Prevalence of overconditioning in mature horses in southwest Virginia during the summer. Journal of Veterinary Internal Medicine. 2012; 26: 1413–1418. 10.1111/j.1939-1676.2012.00995.x 22946995

[2] Potter SJ, Bamford NJ, Harris PA, Bailey SR. Prevalence of obesity and owners' perceptions of body condition in pleasure horses and ponies in south-eastern Australia. Aust Vet J. 2016; 94: 427–432. 10.1111/avj.12506 27785793

[3] Robin CA, Ireland JL, Wylie CE, Collins SN, Verheyen KLP, Newton JR. Prevalence of and risk factors for equine obesity in Great Britain based on owner-reported body condition scores. Equine Veterinary Journal. 2015; 47: 196–201. 10.1111/evj.12275 24735219

[4] Durham AE, Frank N, McGowan CM, Menzies‐Gow NJ, Roelfsema E, Vervuert I, et al ECEIM consensus statement on equine metabolic syndrome. Journal of Veterinary Internal Medicine. 10.1111/jvim.15423 30724412 PMC6430910

[5] Engelhardt Wv, Breves G, Diener M, Gäbel G, editors. Physiologie der Haustiere. 5th ed Stuttgart: Enke Verlag; 2015.

[6] Dougal K, La Fuente G de, Harris PA, Girdwood SE, Pinloche E, Newbold CJ. Identification of a Core Bacterial Community within the Large Intestine of the Horse. PLOS ONE. 2013; 8: e77660 10.1371/journal.pone.0077660 24204908 PMC3812009

[7] Fernandes KA, Kittelmann S, Rogers CW, Gee EK, Bolwell CF, Bermingham EN, et al Faecal microbiota of forage-fed horses in New Zealand and the population dynamics of microbial communities following dietary change. PLoS ONE. 2014; 9: e112846 10.1371/journal.pone.0112846 25383707 PMC4226576

[8] Dougal K, La Fuente G de, Harris PA, Girdwood SE, Pinloche E, Geor RJ, et al Characterisation of the faecal bacterial community in adult and elderly horses fed a high fibre, high oil or high starch diet using 454 pyrosequencing. PLoS ONE. 2014; 9: e87424 10.1371/journal.pone.0087424 24504261 PMC3913607

[9] Morrison PK, Newbold CJ, Jones E, Worgan HJ, Grove-White DH, Dugdale AH, et al The Equine Gastrointestinal Microbiome: Impacts of Age and Obesity. Front Microbiol. 2018; 9 10.3389/fmicb.2018.03017 30581426 PMC6293011

[10] Costa MC, Stämpfli HR, Allen-Vercoe E, Weese JS. Development of the faecal microbiota in foals. Equine Veterinary Journal. 2016; 48: 681–688. 10.1111/evj.12532 26518456

[11] Costa MC, Silva G, Ramos RV, Staempfli HR, Arroyo LG, Kim P, et al Characterization and comparison of the bacterial microbiota in different gastrointestinal tract compartments in horses. The Veterinary Journal. 2015; 205: 74–80. 10.1016/j.tvjl.2015.03.018 25975855

[12] Costa MC, Arroyo LG, Allen-Vercoe E, Stämpfli HR, Kim PT, Sturgeon A, et al Comparison of the faecal microbiota of healthy horses and horses with colitis by high throughput sequencing of the V3-V5 region of the 16S rRNA gene. PLoS ONE. 2012; 7: e41484 10.1371/journal.pone.0041484 22859989 PMC3409227

[13] Blackmore TM, Dugdale A, Argo CM, Curtis G, Pinloche E, Harris PA, et al Strong stability and host specific bacterial community in faeces of ponies. PLoS ONE. 2013; 8: e75079 10.1371/journal.pone.0075079 24040388 PMC3770578

[14] Turnbaugh PJ, Bäckhed F, Fulton L, Gordon JI. Diet-induced obesity is linked to marked but reversible alterations in the mouse distal gut microbiome. Cell Host Microbe. 2008; 3: 213–223. 10.1016/j.chom.2008.02.015 18407065 PMC3687783

[15] Turnbaugh PJ, Hamady M, Yatsunenko T, Cantarel BL, Duncan A, Ley RE, et al A core gut microbiome in obese and lean twins. Nature. 2009; 457: 480–484. 10.1038/nature07540 19043404 PMC2677729

[16] Ley RE, Bäckhed F, Turnbaugh P, Lozupone CA, Knight RD, Gordon JI. Obesity alters gut microbial ecology. Proc Natl Acad Sci U S A. 2005; 102: 11070–11075. 10.1073/pnas.0504978102 16033867 PMC1176910

[17] Ley Ruth E., Turnbaugh Peter J., Klein Samuel, Gordon Jeffrey I. Human gut microbes associated with obesity. Brief communications. Nature. 2006; 441: xi–xi. 10.1038/7093xic 17183309

[18] Bäckhed F, Ding H, Wang T, Hooper LV, Koh GY, Nagy A, et al The gut microbiota as an environmental factor that regulates fat storage. Proc Natl Acad Sci U S A. 2004; 101: 15718–15723. 10.1073/pnas.0407076101 15505215 PMC524219

[19] Rabot S, Membrez M, Bruneau A, Gérard P, Harach T, Moser M, et al Germ-free C57BL/6J mice are resistant to high-fat-diet-induced insulin resistance and have altered cholesterol metabolism. FASEB J. 2010; 24: 4948–4959. 10.1096/fj.10-164921 20724524

[20] Schéle E, Grahnemo L, Anesten F, Hallén A, Bäckhed F, Jansson J-O. The gut microbiota reduces leptin sensitivity and the expression of the obesity-suppressing neuropeptides proglucagon (Gcg) and brain-derived neurotrophic factor (Bdnf) in the central nervous system. Endocrinology. 2013; 154: 3643–3651. 10.1210/en.2012-2151 23892476

[21] Turnbaugh PJ, Ley RE, Mahowald MA, Magrini V, Mardis ER, Gordon JI. An obesity-associated gut microbiome with increased capacity for energy harvest. Nature. 2006; 444: 1027–1031. 10.1038/nature05414 17183312

[22] Duncan SH, Lobley GE, Holtrop G, Ince J, Johnstone AM, Louis P, et al Human colonic microbiota associated with diet, obesity and weight loss. Int J Obes (Lond). 2008; 32: 1720–1724. 10.1038/ijo.2008.155 18779823

[23] Armougom F, Henry M, Vialettes B, Raccah D, Raoult D. Monitoring bacterial community of human gut microbiota reveals an increase in Lactobacillus in obese patients and Methanogens in anorexic patients. PLoS ONE. 2009; 4: e7125 10.1371/journal.pone.0007125 19774074 PMC2742902

[24] Million M, Angelakis E, Maraninchi M, Henry M, Giorgi R, Valero R, et al Correlation between body mass index and gut concentrations of Lactobacillus reuteri, Bifidobacterium animalis, Methanobrevibacter smithii and Escherichia coli. Int J Obes (Lond). 2013; 37: 1460–1466. 10.1038/ijo.2013.20 23459324 PMC3826031

[25] Fleissner CK, Huebel N, Abd El-Bary MM, Loh G, Klaus S, Blaut M. Absence of intestinal microbiota does not protect mice from diet-induced obesity. Br J Nutr. 2010; 104: 919–929. 10.1017/S0007114510001303 20441670

[26] Shepherd ML, Ponder MA, Burk AO, Milton SC, Swecker WS. Fibre digestibility, abundance of faecal bacteria and plasma acetate concentrations in overweight adult mares. J Nutr Sci. 2014; 3: e10 10.1017/jns.2014.8 25191602 PMC4153333

[27] Liu R, Hong J, Xu X, Feng Q, Zhang D, Gu Y, et al Gut microbiome and serum metabolome alterations in obesity and after weight-loss intervention. Nat Med. 2017; 23: 859–868. 10.1038/nm.4358 28628112

[28] Biddle AS, Tomb J-F, Fan Z. Microbiome and Blood Analyte Differences Point to Community and Metabolic Signatures in Lean and Obese Horses. Front Vet Sci. 2018; 5 10.3389/fvets.2018.00225 30294603 PMC6158370

[29] Elzinga SE, Weese JS, Adams AA. Comparison of the fecal Microbiota in Horses with Equine Metabolic Syndrome and metabolically normal Controls fed a similar all-forage diet. Journal of Equine Veterinary Science. 2016; 44: 9–16. 10.1016/j.jevs.2016.05.010

[30] Koliada A, Syzenko G, Moseiko V, Budovska L, Puchkov K, Perederiy V, et al Association between body mass index and Firmicutes/Bacteroidetes ratio in an adult Ukrainian population. BMC Microbiol. 2017; 17: 120 10.1186/s12866-017-1027-1 28532414 PMC5440985

[31] Angelakis E, Armougom F, Million M, Raoult D. The relationship between gut microbiota and weight gain in humans. Future Microbiol. 2012; 7: 91–109. 10.2217/fmb.11.142 22191449

[32] Frape D. Equine nutrition and feeding. 1.The digestive system, the large instestine, products of fermentation. S.11. 4th ed Chichester: Wiley-Blackwell; 2010.

[33] Alford P, Geller S, Richrdson B, Slater M, Honnas C, Foreman J, et al A multicenter, matched case-control study of risk factors for equine laminitis. Preventive Veterinary Medicine. 2001; 49: 209–222. 10.1016/s0167-5877(01)00188-x 11311954

[34] Luthersson N, Mannfalk M, Parkin TDH, Harris P. Laminitis: Risk Factors and Outcome in a Group of Danish Horses. Journal of Equine Veterinary Science. 2017; 53: 68–73. 10.1016/j.jevs.2016.03.006

[35] Wylie CE, Collins SN, Verheyen KLP, Newton JR. Risk factors for equine laminitis: A case-control study conducted in veterinary-registered horses and ponies in Great Britain between 2009 and 2011. The Veterinary Journal. 2013; 198: 57–69. 10.1016/j.tvjl.2013.08.028 24070987

[36] Schedlbauer C, Blaue D, Gericke M, Blüher M, Starzonek J, Gittel C, et al Impact of body weight gain on hepatic metabolism and hepatic inflammatory cytokines in comparison of Shetland pony geldings and Warmblood horse geldings. PeerJ. 2019; 7: e7069 10.7717/peerj.7069 31211018 PMC6557249

[37] Blaue D, Schedlbauer C, Starzonek J, Gittel C, Brehm W, Einspanier A, et al Effects of body weight gain on insulin and lipid metabolism in equines. Domestic Animal Endocrinology. 2019 10.1016/j.domaniend.2019.01.003 31035090

[38] Eiler H, Frank N, Andrews FM, Oliver JW, Fecteau KA. Physiologic assessment of blood glucose homeostasis via combined intravenous glucose and insulin testing in horses. American Journal of Veterinary Research. 2005; 66: 1598–1604. 10.2460/ajvr.2005.66.1598 16261835

[39] Durham AE. Endocrine Disease in Aged Horses. Vet Clin North Am Equine Pract. 2016; 32: 301–315. 10.1016/j.cveq.2016.04.007 27449391

[40] Flachowsky G, Kamphues J, Rodehutscord M, Schenkel H, Staudacher W, Südekum K H, et al Empfehlungen zur Energie- und Nährstoffversorgung von Pferden. 2014.

[41] Verordnung (EG) Nr. 152/2009 der Kommission vom 27. Januar 2009 zur Festlegung der Probenahmeverfahren und Analysemethoden für die amtliche Untersuchung von Futtermitteln. VO 152/2009; 2009.

[42] Carroll CL, Huntington PJ. Body condition scoring and weight estimation of horses. Equine Veterinary Journal; 20: 41–45. 10.1111/j.2042-3306.1988.tb01451.x 3366105

[43] Carter RA, Geor RJ, Burton Staniar W, Cubitt TA, Harris PA. Apparent adiposity assessed by standardised scoring systems and morphometric measurements in horses and ponies. Vet J. 2009; 179: 204–210. 10.1016/j.tvjl.2008.02.029 18440844

[44] Jouany J. Volatile fatty acid and alcohol determination in digestive contents, silage juices, bacterial cultures and anaerobic fermentor contents. Science des Aliments. 1982; 2: 131–144.

[45] Yu Z, Morrison M. Improved extraction of PCR-quality community DNA from digesta and fecal samples. Short technical report. BioTechnique. 2004; 36. 10.2144/04365ST04 15152600

[46] Sadet-Bourgeteau S, Philippeau C, Dequiedt S, Julliand V. Comparison of the bacterial community structure within the equine hindgut and faeces using Automated Ribosomal Intergenic Spacer Analysis (ARISA). Animal. 2014; 8: 1928–1934. 10.1017/S1751731114001943 25075719

[47] Read T, Fortun-Lamothe L, Pascal G, Le Boulch M, Cauquil L, Gabinaud B, et al Diversity and Co-occurrence Pattern Analysis of Cecal Microbiota Establishment at the Onset of Solid Feeding in Young Rabbits. Front. Microbiol. 2019; 10: 973 10.3389/fmicb.2019.00973 31134019 PMC6524096

[48] Rognes T, Flouri T, Nichols B, Quince C, Mahé F. VSEARCH: a versatile open source tool for metagenomics. PeerJ. 2016; 4: e2584 10.7717/peerj.2584 27781170 PMC5075697

[49] Mahé F, Rognes T, Quince C, Vargas C de, Dunthorn M. Swarm: robust and fast clustering method for amplicon-based studies. PeerJ. 2014; 2: e593 10.7717/peerj.593 25276506 PMC4178461

[50] Yatsunenko T, Rey FE, Manary MJ, Trehan I, Dominguez-Bello MG, Contreras M, et al Human gut microbiome viewed across age and geography. Nature. 2012; 486: 222–227. 10.1038/nature11053 22699611 PMC3376388

[51] Chen L, Zhang Y-H, Huang T, Cai Y-D. Gene expression profiling gut microbiota in different races of humans. Sci Rep. 2016; 6 10.1038/srep23075 26975620 PMC4791684

[52] Jansen WL, Geelen SNJ, van der Kuilen J, Beynen AC. Dietary soyabean oil depresses the apparent digestibility of fibre in trotters when substituted for an iso-energetic amount of corn starch or glucose. Equine Veterinary Journal. 2002; 34: 302–305. 10.2746/042516402776186074 12108752

[53] Bush JA, Freeman DE, Kline KH, Merchen NR, Fahey GC. Dietary fat supplementation effects on in vitro nutrient disappearance and in vivo nutrient intake and total tract digestibility by horses. J Anim Sci. 2001; 79: 232–239. 10.2527/2001.791232x 11204705

[54] Neumann AP, Suen G. The Phylogenomic Diversity of Herbivore-Associated Fibrobacter spp. Is Correlated to Lignocellulose-Degrading Potential. mSphere. 2018; 3 10.1128/mSphere.00593-18 30541780 PMC6291624

[55] Ragnarrson S., Jansson A. Comparison of grass haylage digestibility and metabolic plasma profile in Icelandic and standard bred horses. Journal of animal physiology and animal nutrition. 2010. 10.1111/j.1439-0396.2010.01049.x 20796073

[56] Steelman SM, Chowdhary BP, Dowd S, Suchodolski J, Janečka JE. Pyrosequencing of 16S rRNA genes in fecal samples reveals high diversity of hindgut microflora in horses and potential links to chronic laminitis. BMC Vet Res. 2012; 8: 231 10.1186/1746-6148-8-231 23186268 PMC3538718

[57] Jensen RB, Broker C, Knudsen KEB, Tauson AH. A comparative study of the apparent total tract digestibility of carbohydrates in Icelandic and Danish warmblood horses fed two different haylages and a concentrate consisting of sugar beet pulp and black oats. Arch Anim Nutr. 2010; 64: 343–356. 10.1080/1745039X.2010.504606 21114231

[58] Shepherd ML, Swecker WS, Jensen RV, Ponder MA. Characterization of the fecal bacteria communities of forage-fed horses by pyrosequencing of 16S rRNA V4 gene amplicons. FEMS Microbiol Lett. 2012; 326: 62–68. 10.1111/j.1574-6968.2011.02434.x 22092776

[59] Grimm P, Philippeau C, Julliand V. Faecal parameters as biomarkers of the equine hindgut microbial ecosystem under dietary change. Animal. 2017; 11: 1136–1145. 10.1017/S1751731116002779 28065211

[60] Julliand V, Grimm P. HORSE SPECIES SYMPOSIUM: The microbiome of the horse hindgut: History and current knowledge. J Anim Sci. 2016; 94: 2262–2274. 10.2527/jas.2015-0198 27285903

