# Supplementary material for: Changes in the faecal microbiota of horses and ponies during a two-year body weight gain programme
Source: PLoS One. 2020 Mar 19;15(3):e0230015. doi: 10.1371/journal.pone.0230015 (PMC7082044; doi:10.1371/journal.pone.0230015)
Supplement: S1 Table — Data shown as %. (DOCX) [file pone.0230015.s001.docx]

**S1 Table:** Coefficient of variation for the nutrient intake in horses and ponies two months prior to sampling point t1, t2, t3 and in between the three sampling points (CP: crude protein, CL: crude lipid, CF: crude fibre, aNDFom: neutral detergent fibre). Data shown as %.

| Time | Hay | Starch | CP | CL | CF | aNDFom | sugar |
| --- | --- | --- | --- | --- | --- | --- | --- |
| -t1 | 8.58 | 11.8 | 13.3 | 10.9 | 9.13 | 9.75 | 8.75 |
| -t2 | 4.64 | 11.2 | 6.47 | 9.16 | 10.8 | 4.66 | 5.38 |
| -t3 | 6.41 | 11.6 | 8.56 | 11.9 | 8.00 | 9.97 | 6.84 |
| -t1-t3 | 10.1 | 18.5 | 14.0 | 22.0 | 10.7 | 12.0 | 12.4 |
| -t1-t2 | 10.8 | 18.6 | 15.5 | 22.2 | 11.5 | 11.2 | 9.69 |
| -t2-t3 | 6.37 | 12.9 | 10.9 | 12.3 | 10.7 | 10.8 | 9.37 |
