# Supplementary material for: Changes in the faecal microbiota of horses and ponies during a two-year body weight gain programme
Source: PLoS One. 2020 Mar 19;15(3):e0230015. doi: 10.1371/journal.pone.0230015 (PMC7082044; doi:10.1371/journal.pone.0230015)
Supplement: S2 Table — (DOCX) [file pone.0230015.s002.docx]

**S2 Table**: Relative abundance of Phyla in the faeces of horses and ponies with an overall median relative abundance over 0.4 % at the three sampling points presented as medians and 25/ 75 percentiles in brackets.

| **Phylum** | **Breed** | **t1** | **t2** | **t3** |
| --- | --- | --- | --- | --- |
| Firmicutes | Horses | 55.7  (52.3/59.8) | 57.2  (55.5/62.2) | 58.5  (55.5/62.9) |
|  | Ponies | 54.0^ab^  (51.9/62.9) | 58.5^a^  (53.2/63.8) | 62.8^b^  (57.7/65.7) |
| Bacteroidetes | Horses | 39.0  (35.0/40.5) | 37.2  (34.0/40.4) | 35.0  (32.7/36.5) |
|  | Ponies | 40.9  (33.1/43.6) | 36.6  (29.4/42.9) | 32.1  (28.9/38.3) |
| Spirocheates | Horses | 3.16  (1.87/3.96) | 1.72  (1.65/2.59) | 2.39  (1.73/3.16) |
|  | Ponies | 2.21  (1.70/3.65) | 1.89  (1.75/2.18) | 1.78  (1.34/2.28) |
| Fibrobacteres | Horses | 1.90^a^  (1.05/2.45) | 0.98^b^  (0.85/1.29) | 1.11^b#^  (0.59/1.84) |
|  | Ponies | 0.78  (0.51/2.31) | 0.79  (0.65/1.70) | 0.38^*^  (0.09/0.88) |
| Proteobacteria | Horses | 0.34  (0.31/0.69) | 0.61^#^  (0.52/0.92) | 0.52  (0.43/0.99) |
|  | Ponies | 0.60^a^  (0.37/0.85) | 1.14^b*^  (0.81/1.45) | 0.43^a^  (0.27/0.70) |
| Actinobacteria | Horses | 0.37^a^  (0.30/0.49) | 0.34^ab^  (0.30/0.43) | 0.88^b^  (0.54/1.31) |
|  | Ponies | 0.33^a^  (0.31/0.49) | 0.39^a^  (0.33/0.59) | 0.77^b^  (0.67/1.25) |

a, b medians with different subscript letters differ significantly within a row (p < 0.05)

*, # medians with different subscript symbols differ significantly within a column (p < 0.05)
