# Supplementary material for: Changes in the faecal microbiota of horses and ponies during a two-year body weight gain programme
Source: PLoS One. 2020 Mar 19;15(3):e0230015. doi: 10.1371/journal.pone.0230015 (PMC7082044; doi:10.1371/journal.pone.0230015)
Supplement: S3 Table — (DOCX) [file pone.0230015.s003.docx]

**S3 Table**: Relative abundance of Classes in the faeces of horses and ponies with an overall median relative abundance over 0.4 % at the three sampling points presented as median and 25/ 75 percentiles in brackets.

| **Class** | **Breed** | **t1** | **t2** | **t3** |
| --- | --- | --- | --- | --- |
| Clostridia | Horses | 54.4  (51.9/59.7) | 56.1  (54.2/59.5) | 56.7  (53.6/60.7) |
|  | Ponies | 53.3  (51.0/62.0) | 57.5  (51.9 /62.2) | 60.2  (56.5/63.4) |
| Bacteroidia | Horses | 39.0  (35.0/40.5) | 37.2  (34.0/40.4) | 35.0  (32.7/36.5) |
|  | Ponies | 40.9  (33.1/43.6) | 36.6  (29.4/42.9) | 32.1  (29.0/38.3) |
| Spirochaetia | Horses | 3.15  (1.78/3.92) | 1.70  (1.61/2.46) | 2.39  (1.72/3.13) |
|  | Ponies | 1.90  (1.58/2.90) | 1.80  (1.58/1.99) | 1.70  (0.94/2.25) |
| Fibrobacteria | Horses | 1.90^a^  (1.06/2.45) | 0.98^b^  (0.85/1.29) | 1.11^b#^  (0.59/1.84) |
|  | Ponies | 0.78  (0.51/2.31) | 0.80  (0.65/1.70) | 0.38*  (0.09/0.88) |
| Bacilli | Horses | 0.39^a^  (0.23/0.66) | 0.42^a^  (0.27/0.61) | 1.21^b^  (0.81/2.37) |
| Coriobacteriia | Horses | 0.37^a^  (0.29/0.49) | 0.34^ab^  (0.30/0.43) | 0.80^b^  (0.52/1.29) |
|  | Ponies | 0.33^a^  (0.30/0.49) | 0.39^a^  (0.33/0.59) | 0.77^b^  (0.67/1.25) |
| Negativicutes | Horses | 0.31  (0.12/0.38) | 0.57  (0.41/0.70) | 0.49  (0.39/0.57) |
|  | Ponies | 0.32^a^  (0.27/0.64) | 0.81^b^  (0.41/1.24) | 0.39^a^  (0.23/0.84) |

a, b medians with different subscript letters differ significantly within a row (p < 0.05)

*, # medians with different subscript symbols differ significantly within a column (p < 0.05
