# Supplementary material for: Changes in the faecal microbiota of horses and ponies during a two-year body weight gain programme
Source: PLoS One. 2020 Mar 19;15(3):e0230015. doi: 10.1371/journal.pone.0230015 (PMC7082044; doi:10.1371/journal.pone.0230015)
Supplement: S4 Table — (DOCX) [file pone.0230015.s004.docx]

**S4 Table**: Relative abundance of Orders in the faeces of horses and ponies with an overall median relative abundance over 0.4 % at the three sampling points presented as median and 25/ 75 percentiles in brackets.

| **Order** | **Breed** | **t1** | **t2** | **t3** |
| --- | --- | --- | --- | --- |
| Clostridiales | Horses | 54.4  (52.0/59.7) | 56.1  (54.2/59.5) | 56.7  (53.6/60.7) |
|  | Ponies | 53.3  (51.0/62.0) | 57.5  (51.9/62.2) | 60.2  (56.5/63.4) |
| Bacteroidales | Horses | 39.0  (35.0/40.5) | 37.2  (33.8/40.4) | 35.0  (32.6/36.4) |
|  | Ponies | 40.9  (33.1/43.6) | 36.6  (29.4/42.9) | 32.1  (29.0/ 38.3) |
| Spirochaetales | Horses | 3.15  (1.78/3.92) | 1.70  (1.61/2.46) | 2.39  (1.72/3.13) |
|  | Ponies | 1.90  (1.58/2.90) | 1.79  (1.58/2.00) | 1.70  (0.94/2.25) |
| Fibrobacterales | Horses | 1.90  (1.06/2.45) | 0.98  (0.85/1.29) | 1.11^#^  (0.59/1.84) |
|  | Ponies | 0.78^a^  (0.51/2.31) | 0.80^b^  (0.65/1.70) | 0.38^a*^  (0.09/0.88) |
| Lactobacillales | Horses | 0.39  (0.23/0.66) | 0.42  (0.27/0.61) | 1.23  (0.78/ 2.35) |
| Coriobacteriales | Horses | 0.37^a^  (0.30/0.49) | 0.34^ab^  (0.29/0.43) | 0.80^b^  (0.52/1.29) |
|  | Ponies | 0.33^a^  (0.30/0.49) | 0.39^a^  (0.33/0.60) | 0.77^b^  (0.67/1.25) |
| Selenomonadales | Horses | 0.31  (0.12/0.38) | 0.57  (0.41/0.70) | 0.49  (0.39/0.57) |
|  | Ponies | 0.32^a^  (0.27/0.64) | 0.8^b^  (0.41/1.24) | 0.39^a^  (0.23/0.84) |

a, b medians with different subscript letters differ significantly within a row (p < 0.05)

*, # medians with different subscript symbols differ significantly within a column (p < 0.05)
