# Supplementary material for: Changes in the faecal microbiota of horses and ponies during a two-year body weight gain programme
Source: PLoS One. 2020 Mar 19;15(3):e0230015. doi: 10.1371/journal.pone.0230015 (PMC7082044; doi:10.1371/journal.pone.0230015)
Supplement: S5 Table — (DOCX) [file pone.0230015.s005.docx]

**S5 Table**: Relative abundance of Families in the faeces of horses and ponies with an overall median relative abundance over 0.4 % at the three sampling points presented as median and 25/ 75 percentiles in brackets.

| **Family** | **Breed** | **t1** | **t2** | **t3** |
| --- | --- | --- | --- | --- |
| Ruminococcaceae | Horses | 25.0^a^  (22.5/29.1) | 24.2^a^  (22.7/31.9) | 21.4^b#^  (19.0/22.6) |
|  | Ponies | 24.5  (23.9/32.2) | 27.2  (24.1/33.9) | 25.2*  (20.9/26.1) |
| Lachnospiraceae | Horses | 23.9^a^  (20.5/25.6) | 22.9^ab^  (20.8/26.4) | 31.3^b^  (25.9/32.5) |
|  | Ponies | 22.7  (20.7/23.3) | 22.7  (20.8/25.0) | 26.8  (24.0/28.6) |
| p-251-o5 | Horses | 14.1  (10.6/15.5) | 12.4  (8.88/ 13.8) | 11.5  (8.59/13.9) |
|  | Ponies | 12.9  (6.38/13.8) | 9.96  (7.23/11.9) | 8.55  (3.08/ 10.3) |
| Prevotellaceae | Horses | 10.8  (8.12/11.9) | 9.97  (7.55/11.7) | 11.6  (10.1/ 13.0) |
|  | Ponies | 8.34  (6.46/9.94) | 10.4  (6.83/13.2) | 9.88  (5.34/12.3) |
| Rikenellaceae | Horses | 7.15  (5.33/7.65) | 5.63  (4.19/6.39) | 4.80^#^  (4.18/6.17) |
|  | Ponies | 7.40^ab^  (4.51/8.17) | 5.58^a^  (4.77/5.93) | 6.80^b*^  (5.56/9.50) |
| F082 | Horses | 2.66^a#^  (1.81/2.84) | 4.46^a^  (2.13/9.44) | 1.50^b#^  (1.10/1.88) |
|  | Ponies | 6.34^*^  (4.09/9.94) | 5.67  (3.29/7.00) | 5.16^*^  (2.65/5.74) |
| Christensenellaceae | Horses | 2.95  (2.55/3.25) | 3.29  (2.99/3.68) | 3.33  (2.99/3.96) |
|  | Ponies | 3.31  (2.96/3.71) | 4.30  (3.33/4.85) | 3.87  (3.08/6.95) |
| Spirochaetaceae | Horses | 3.15  (1.78/3.92) | 1.70  (1.62/2.46) | 2.39  (1.72/3.13) |
|  | Ponies | 1.90  (1.58/2.90) | 1.80  (1.58/2.00) | 1.70  (0.94/2.25) |
| Family XIII | Horses | 1.91  (1.67/2.27) | 1.91  (1.85/2.42) | 2.01  (1.64/2.86) |
|  | Ponies | 1.80  (1.64/1.97) | 2.15  (1.90/2.46) | 3.09  (1.48/4.26) |
| Bacteroidales UCG-001 | Horses | 1.14  (0.94/1.84) | 0.90  (0.62/1.39) | 0.60^#^  (0.52/0.93) |
|  | Ponies | 1.22  (1.02/1.64) | 0.90  (0.75/1.13) | 1.21^*^  (1.03/142) |
| Fibrobacteraceae | Horses | 1.90^a^  (1.06/2.45) | 0.98^b^  (0.85/1.29) | 1.11^b#^  (0.59/1.84) |
|  | Ponies | 0.78  (0.51/2.31) | 0.80  (0.65/1.70) | 0.38^*^  (0.09/0.88) |
| Paludibacteraceae | Horses | 0.68  (0.26/0.81) | 0.35  (0.19/1.15) | 0.26  (0.13/0.67) |
|  | Ponies | 0.44  (0.17/1.66) | 0.57  (0.34/1.07) | 0.12  (0.05/ 0.79) |
| Muribaculaceae | Horses | 0.75  (0.61/1.17) | 1.55  (1.10/1.74) | 1.24  (0.84/2.81) |
|  | Ponies | 0.93^a^  (0.47/1.93) | 1.62^b^  (1.13/2.64) | 1.14^a^  (0.72/1.57) |
| Bacteroidales RF16 group | Horses | 0.81^a^  (0.59/1.00) | 0.45^ab^  (0.28/0.63) | 0.29^b^  (0.13/0.50) |
|  | Ponies | 0.62^a^  (0.36/0.89) | 0.40^ab^  (0.35/0.49) | 0.17^b^  (0.14/0.28) |
| Veillonellaceae | Horses | 0.31  (0.12/0.38) | 0.57  0.41/0.70) | 0.49  (0.39/0.57) |
|  | Ponies | 0.32^a^  (0.27/0.64) | 0.81^b^  (0.41/1.24) | 0.39^a^  (0.23/0.84) |

a, b medians with different subscript letters differ significantly within a row (p < 0.05)

*, # medians with different subscript symbols differ significantly within a column (p < 0.05
